# Telomere Length Changes during Critical Illness: A Prospective, Observational Study

**DOI:** 10.3390/genes10100761

**Published:** 2019-09-27

**Authors:** Benjamin Zribi, Orit Uziel, Meir Lahav, Ronit Mesilati Stahy, Pierre Singer

**Affiliations:** 1 Department of Anesthesiology, Rabin Medical Center, Campus Beilinson, Petah Tikva 49100, Israel; 2 The Felsenstein Medical Research Center, Rabin Medical Center, Campus Beilinson, Petah Tikva and the Sackler School of Medicine, Tel Aviv University, Petah Tikva 49100, Israel; Oritu@clalit.org.il; 3 Institute of Hematology, Davidoff Cancer Center, Rabin Medical Center and the Sackler School of Medicine, Tel Aviv University, Petah Tikva 49100, Israel; mlahav@tauex.tau.ac.il; 4 The Felsenstein Medical Research Center, Rabin Medical Center, Campus Beilinson, Petah Tikva 49100, Israel; ronit_mesilati@walla.co.il; 5 Department of General Intensive Care and Institute for Nutrition Research, Rabin Medical Center, Campus Beilinson and the Sackler School of Medicine, Tel Aviv University, Petah Tikva 49100, Israel; pierre.singer@gmail.com

**Keywords:** telomeres, intensive care, acute illness, sepsis, white blood cells

## Abstract

Objective: evaluation of telomere length change in acutely ill adult patients. Design: Blood samples were drawn on the first and seventh day of intensive care unit (ICU) stay to assess telomere length using a polymerase chain reaction (PCR)-based technique. Demographic data collected included age, weight, admission diagnosis, baseline laboratory values (pH, C- reactive protein (CRP), serum albumin level, white blood cell count (WBC) count, platelet count), and baseline SOFA and APACHE II scores. Additional data collected during the ICU stay included a repeated WBC count, the presence of positive blood cultures and outcome data, including death in the ICU or following discharge, whether ventilated or not at ICU discharge, and destination following discharge, i.e., medical ward or rehabilitation. Setting: General ICU in tertiary hospital. Patients: Forty patients admitted to the ICU within 72 h of hospital admission suffering from an acute illness were included in this prospective, observational study. Main results: Of the 40 patients studied, telomere shortening was noted in 21, telomere lengthening in 11, and no significant change in the other eight. The age of patients demonstrating telomere shortening was statistically significantly younger (45.4 vs. 61.5 years, *p* < 0.023) compared to those showing increased telomere length. In addition, a significant correlation was observed between the difference in telomere length and the corresponding difference in WBC count (telomere shortening was associated with a decreased WBC count and vice versa). A trend toward shortening was seen in patients with sepsis (*p* = 0.07). No significant correlations were found for any other demographic or outcome parameter and changes in telomere length. Conclusion: Changes in telomere length, both shortening and lengthening, were evident in the acute setting, but no associations between such changes with outcome were noted. Further studies in more homogeneous groups of patients appear to be warranted.

## 1. Introduction

Critically ill patients hospitalized in the intensive care unit (ICU) typically develop an inflammatory, hypermetabolic state defined as the acute phase, which is associated with severe metabolic stress [1]. A common example is sepsis, which is defined as life-threatening organ dysfunction due to a dysregulated host response to infection [2], which may result in a complex proinflammatory and coagulant response [3,4,5,6,7]. This state, referred to as systemic inflammatory response syndrome (SIRS), is typically associated with the production of various labile reactive species, including gaseous mediators as well as reactive oxygen species, which play a central role in the pathophysiologic events characterizing the inflammatory response. Importantly, oxidative stress is an important pathophysiological trigger that may lead to DNA damage [8].Telomeres, located at the ends of each chromosome, are crucial genomic structures that protect chromosomes from decay, thus maintaining genomic integrity. Telomere structures are composed of 1000–2000 repeats of TTAGGG sequences in humans, coated by six-protein complexes called shelterins. These complexes protect telomeres from being recognized by cellular DNA repair mechanisms that otherwise may define them as double-strand DNA breaks that need to be repaired, resulting in end-to-end fusions and chromosomal instabilities. These structures normally undergo attrition during each cell division, so that processes such as growth and advancing age are associated with telomere shortening. The mean telomere shortening is 20–200 base pairs during each cell cycle until a critical point is reached, whereupon senescence and apoptosis of the cell follow [9]. Many biochemical, environmental, and genetic factors are currently being studied to assess their impact on telomere length. Thus, correlations have been observed between leukocyte telomere length and common age-related disorders, including atherosclerotic disease, heart failure, cancer, type 2 diabetes mellitus, and dementia, especially Alzheimer’s disease [9,10,11,12,13,14]. Oxidative damage is thought to be the major environmental factor that accelerates telomere shortening, in vitro as well as in vivo [15], and shorter telomeres have been shown to correlate with increased levels of inflammation [16]. In this regard, chronic stress has been demonstrated to be an important factor contributing to telomere attrition in both animal models and cell cultures [17]. Another large study by Van den Berghe et al. [18] was performed with a critically ill pediatric population. This study included many patients (*n* = 644) and showed a correlation between telomere attrition of 6% and early parenteral nutrition administration. Inflammation is highly linked to telomere attrition as well. A recent study observed a highly variable rate of change in telomere length of PBMCs with a relatively slow average rate of telomere shortening (−16 bp/year). This shortening was associated with a significant increase with age in three inflammation-related cytokines (interferon gamma, IL-6, and IL-10) and in anti-CMV IgG titer, which also varied widely across individuals. A positive correlative was observed between changes among different inflammatory cytokines. Since no correlation was found between telomere attrition, cytokine levels, and anti-CMV IgG, the authors concluded that age-related trajectories of telomere attrition, elevated circulating inflammatory cytokines, and anti-CMV IgG are independent, pointing to the fact that immune aging processes are complex and vary across individuals [19].

Findings from a recent small study of septic patients (*n* = 9) showed that significant reductions in telomere length may also occur over short time periods, namely over one week [20]. However, the correlation between changes of leukocyte telomere length in this acute setting of critically ill, metabolically stressed patients and outcome has not been evaluated to date. In this prospective study, we aimed to investigate any changes in telomere length occurring in the acute setting of a larger cohort of critically ill patients and study any association between such changes and the outcome.

## 2. Materials and Methods

### 2.1. Study Population

This prospective, observational study was performed in the 14-bed, general intensive care department of the Rabin Medical Center, Campus Beilinson and was approved by the local ethics committee review board, which waived the need for consent. The study was performed over the period of October 2017 until March 2018. Patients who met the following criteria were included in the study: recent hospital admission (i.e., <72 h previously), a medical condition requiring ICU admission and a predicted length of ICU stay ≥5 days. Patients were excluded if their age was <18 or >75 years and if they were pregnant or in the immediate postpartum period.

Ethics approval: The study protocol was approved by Rabin Medical Center’s local ethics committees and informed consent was waived. Approval number rmc-0319-11.

### 2.2. Data Collection

Demographic data collected on admission included age, weight, main admission diagnosis, and whether the patient was ventilated before admission to the ICU, and SOFA and APACHE II scores were calculated. Baseline laboratory data collected included pH, C-reactive protein (CRP-mg/dL), serum albumin (g/L), white blood cell count (WBC, K/mcL), and platelet count (K/mcL). Additional data collected during the ICU admission included the presence of any positive blood cultures and the WBC count on the day of the second blood sample analysis. Outcome data included death in the ICU or following discharge (up to six months), whether the patient was ventilated at ICU discharge and destination following discharge, i.e., medical ward or rehabilitation.

Availability of Data and Materials: The datasets used and/or analyzed during the current study are available from the corresponding author upon a reasonable request.

### 2.3. Telomere Length Analysis

Telomere length was determined from two blood samples: the initial sample drawn in the first 72 h after hospitalization and the second after <5 or >14 days. For patients discharged before day 5, a repeat sample was drawn and analyzed on the discharge day. The blood sample processing was as follows: 5 mL of blood was collected and the red blood cells (RBC) were lysed using the RBC lysis solution (Biological Industries, Beit Haemek, Israel). Isolation of genomic DNA was performed by using the DNA isolation kit for mammalian blood (Roche, Mannheim, Germany). Briefly, DNA was isolated by the salting-out procedure, washed, and precipitated by isopropanol. The DNA was resuspended in polymerase chain reaction (PCR)-grade water. The DNA concentration was measured by using the NanoDrop device (Thermo Fisher, Waltham, MA, USA).

DNA samples were analyzed for telomere length according to the method of Cawthon (2009) [21], with slight modifications. Each DNA sample was analyzed by two sets of primers detailed below, one for telomere length analysis and one for a reference gene analysis (human hemoglobin). The primers were diluted to 100 µM in PCR grade water and then to 10 µM. DNA samples were diluted to 2.5 ng/µL in PCR grade water. The primers sequences are shown below:

**telc:** TGTTAGGTATCCCTATCCCTATCCCTATCCCTATCCCTAACA

**telg**: ACACTAAGGTTTGGGTTTGGGTTTGGGTTTGGGTTAGTGT

**hbgd:** GCCCGGCCCGCCGCGCCCGTCCCGCCGGAGGAGAAGTCTGCCGTT

**hbgu:** GGCGGCGGGCGGCGCGGGCTGGGCGGCTTCATCCACGTTCACCTTG

Reaction PCR were processed as follows: 50 °C for 2 min, 95 °C for 5 min, a single cycle of 94 °C for 15 s, 49 °C for 15 s; 40 cycles of: 94 °C for 15 s, 62 °C for 10 s and a final stage of: 74 °C for 15 s. All reactions were performed using a Step One device (ABI, Waltham, MA, USA).

As a quality control for the PCR method, we used other blood samples from healthy individuals (Table S1) and obtained no variations among different independent runs (not shown). In addition, we normalized the results of the PCR reactions by running a specific DNA sample throughout the various runs.

A significant change in telomere length was defined as a 15% difference in length between the two measurements in order to take into account possible variations in telomere length [22].

### 2.4. Statistical Analysis

Continuous variables are shown as the mean ± standard deviation(SD), while categorical variables are shown as number (*n*) (%). Analysis of variance was used to compare the value of continuous variables between telomere groups and the chi-square test was used for categorical variables. Survival was assessed using the Kaplan‒Meier survival analysis, with the log-rank test. Pearson correlation was used to assess associations between continuous variables. A two-sided *p* value < 0.05 was considered statistically significant (SAS Software, Version 9.4, 100 SAS Campus Drive Cary, NC 27513-2414, USA).

## 3. Results

**Figure 1:**
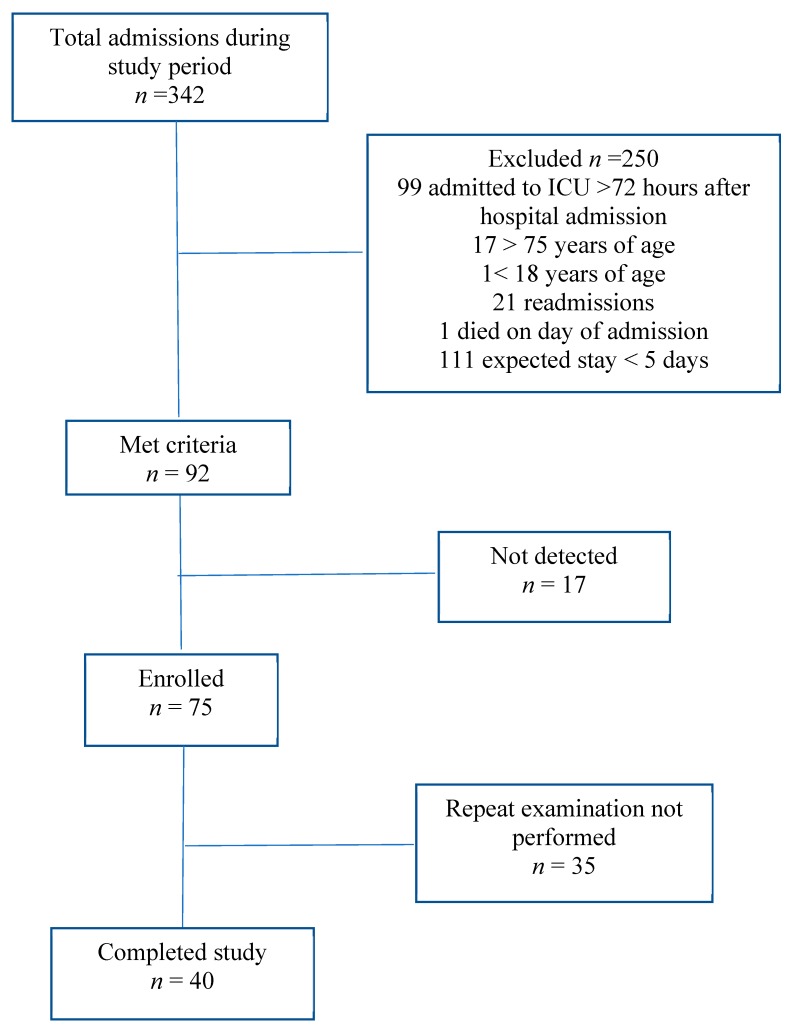
Flowchart of patient selection; see details in text. *n-number of patients*, ICU-intensive care unit.

The patient selection process is shown in Figure 1.

In total, 75 patients were included in the study. Of these, a repeated sample was obtained from 40 patients (after a mean of 7.2 ± 2.5 days), while the remaining 35 patients with only a single measurement were excluded. Patient demographics and baseline laboratory values are shown in Table 1.

The mean age of the patients was 50.6 (±18.8) years and the mean body mass index (BMI) was 28 (±6.9). Most patients were male (78%), reflecting the gender mix of the ICU. The mean APACHE II and SOFA scores on admission were 22.8 ± 6.8 and 9.6 ± 3.32, respectively. Admission diagnoses were medical in 55% of cases, including renal failure, chronic obstructive pulmonary disease (COPD), sepsis, cerebrovascular accident (CVA), encephalitis, liver failure, hyponatremia, post-cardiopulmonary resuscitation (CPR), and surgical in 45% of cases including trauma, upper gastrointestinal (GI) bleeding, liver transplant, bowel perforation, and necrotizing fasciitis.

Regarding changes in telomere length, 32 (80%) patients demonstrated a significant change in length, of whom 11 (27.5%) showed an increase (from 23.7% to 100% of baseline) and 21 (52.5%) showed a decrease (69.5% to 17.6% of baseline). Eight patients did not show the threshold change of 15%; a detailed description of telomere length is given in Table 2.

No statistically significant correlation was found in the univariate analysis with telomere length and any of the following variables: BMI, APACHE II score on admission, SOFA score on admission, main diagnosis, diagnostic group (medical vs. surgical), and any outcome variable (Table 3).

**Figure 2:**
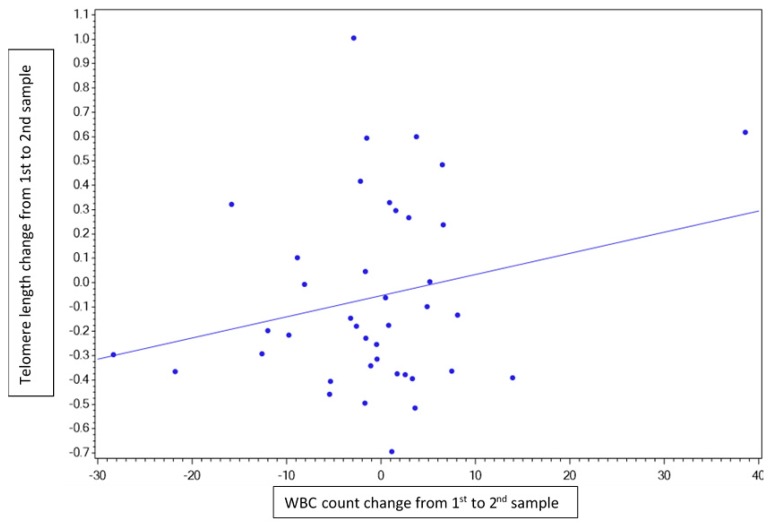
Pearson correlation graph. This figure demonstrates a weak link between telomere dynamics (difference in length between first and second measurement) and white blood cells (WBC) count dynamics (subtracted from WBC sampling on the day of telomere sampling).

**Figure 3:**
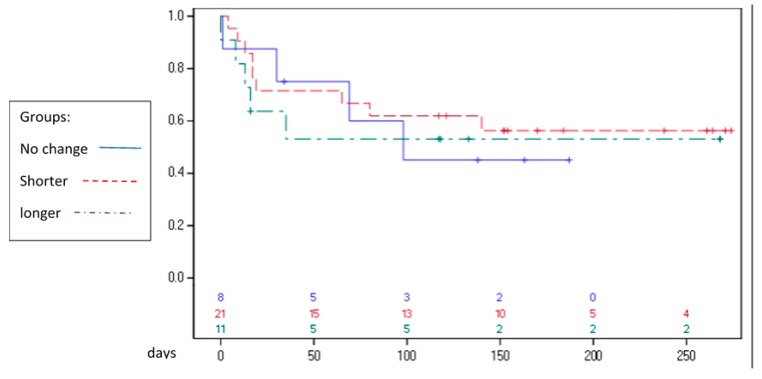
Kaplan‒Meier curve. Follow-up of mortality from admission up to six months after study completion. Note that some patients were enrolled prior to others, hence the differential follow-up time.

The age of patients demonstrating telomere shortening was statistically significantly younger (45.4 years vs. 61.5 years) compared with those showing increased telomere length (*p* < 0.023). A correlation was observed between a change in telomere length and a corresponding change in the repeated WBC count (i.e., telomere shortening was associated with a decreased WBC count on the second examination and telomere lengthening was associated with an increased WBC count on the second examination) with a Pearson correlation of 0.23 (Figure 2). There was no correlation between mortality and telomere length difference in any direction, as shown in Figure 3.

## 4. Discussion 

In this study, we demonstrated that significant changes in telomere length, both increases and decreases, occurred over a short time period (mean 7.2 ± 2.5 days) in adult, critically ill patients in the ICU. However, we were unable to demonstrate a significant correlation between telomere length changes and any outcome measure.

Telomere shortening has consistently been shown to occur in response to exogenous stress, mainly by oxidative damage [23,24]. Thus, for example, patients with Fanconi anemia (FA) suffer from a dysfunctional cellular response to the metabolism of oxygen, and fibroblasts from these patients demonstrate shorter telomeres and higher rates of telomere attrition compared to normal age-matched controls [13,24,25]. The causative connection between stress, mostly oxidative, and telomere shortening has also been shown in several other conditions, such as cyclosporine-induced nephrotoxicity [26] and in patients with hepatitis C [13]. Interestingly, a decrease in telomere shortening in human skin keratinocytes has been demonstrated following exposure to the anti-oxidative effects of pro-vitamin C [27]. Cells in the human brain also showed slowed telomere shortening after exposure to phosphorylated alpha tocopherol [28]. In an attempt to understand the mechanisms underlying oxidative stress-induced telomere shortening, Kawanishi et al. [29] followed the formation of site-specific DNA damage induced by Ultraviolet A (UVA) irradiation in the presence of endogenous photosensitizers on telomeric oligonucleotide (TTAGGG). The authors concluded that the formation of 8-oxodG at the GGG triplet in the telomere sequence induced by oxidative stress could participate in acceleration of telomere shortening [30,31].

Our findings confirm that telomere length may be altered significantly in patients with acute stress. The fact that both shortening (in the majority) and lengthening were observed suggests that multiple mechanisms may be involved in telomere length regulation in this setting and presumably through different mechanisms. Thus, while all critically ill patients may be considered to suffer from “acute metabolic stress,” this definition may be too encompassing. Our finding of a correlation between changes in telomere length and the white blood cell count may suggest that acute stress leads to a free oxygen radical “hit” on the circulating white cells. It is unknown if this hit also affects the newly produced white blood cells. Our study also demonstrates a trend toward a correlation between telomere shortening and a diagnosis of sepsis. In this regard, Oliveira et al. demonstrated that induction of endotoxemia in mice resulted in significant telomere shortening in the spleen and kidney, while blood cells from patients who progressed to sepsis also exhibited a statistically significant reduction of telomere length [20]. This was associated with a concomitant increase in oxidative stress, which the authors hypothesized may contribute to telomere shortening in sepsis. In this regard, the shortening observed in our study cannot be explained by replicative senescence, as normal telomere attrition in healthy individuals previously measured by us is about 50 bp/year [32].

Interestingly, and in contrast to the previous study with septic patients, telomere lengthening was noted in one of our patients. The mixed behavior of telomere length in other groups of patients may imply that ongoing oxidative stress, such as typically occurs in severe sepsis, is required for telomere shortening, while transient stress, such as may be seen following trauma, may allow the white cells to recover.

Another possible explanation for the lengthening of telomeres obtained in our setting may be related to the ALT (alternative lengthening of telomeres) mechanism by which telomeres are elongated by homologous recombination-based mechanism. Whether this hypothesis is correct and why ALT occurred in the specific patients is not known at the moment and may warrant further study.

Unfortunately, to date there is no consensus regarding the definition of significant attrition. We used a cutoff of 15%, which has been used by others [20]. This is higher than what was used in the largest study to date by Van den Berghe et al., who used a cutoff of 6% to describe significant attrition [18]. Further studies are required to provide a possible optimal cutoff of significant changes in telomere length.

There are several limitations to this study. Firstly, although our cohort is the largest adult cohort to examine telomere length dynamics under acute stress studied to date and included those with a high APACHE score and limited inclusion to the first 14 days, i.e., the acute phase, the cohort included a heterogeneous population, so any significant correlations may be difficult to detect. Secondly, although it is very simple and quick, the method chosen for measuring telomere length has several pitfalls. Since the sequence of telomeres is a repetitive one [(TTAGGG)n] and it is a PCR-based methodology, the primers used for the PCR reaction may attach to several places in the telomere. Therefore, the PCR product that is formed includes a population of telomere-based products of various lengths, of which we calculate the average. In spite of this, there is a clear correlation between the actual lengths of telomeres (measured in other methods including Southern blot) and the signal obtained by the PCR method. Logically, there is a direct relationship between the two: the longer the telomere is, the higher the PCR signal will be. To counteract this problem, we conducted all PCR reactions in quadraplexes and considered only results that were repeated at least twice. Importantly, the scientist who established this widely used method has proved the validity of it by comparing it to the Southern blot method (considered as the gold standard for telomere length measuring).

The other point is related to the fact that changes in telomere length can be due to a shift in leukocyte subpopulations. Therefore, an increase in total telomere length may stem from an increase in monocytes or B cells with longer telomeres. However, during a short hospitalization in the ICU the leukocyte subpopulations are not expected to change dramatically, so we have decided to ignore this issue here.

Finally, this was an observational, single-center study.

## 5. Conclusions 

We have shown that changes in telomere length may occur during acute stress. However, we were unable to demonstrate a correlation between telomere behavior and any outcome measure studied, which may be related to the heterogeneity of the cohort. Additional studies focusing on specific population groups will be required to further elucidate our findings.

## Figures and Tables

**Table 1:** Characteristics and laboratory data of patients admitted to the ICU unit, mean (± SD (standard deviation)).

Characteristic	*n* = 40
Age (years)	50.6 (±18.8)
BMI (kg/m^2^)	28 (±6.9)
Gender (male), *n* (%)	31 (78%)
APACHE II score	22.8 (±6.8)
Ventilated on admission, *n* (%)	37 (93%)
WBC count on admission (K/mcL)	12 (±5.7)
Platelets count on admission (K/mcL)	190 (±96)
CRP on admission (mg/dL)	14.7 (±12.6)
Main diagnosis	
Sepsis	8 (20%)
Respiratory	5 (12.5%)
Trauma	14 (35%)
Organ Transplant	3 (7.5%)
Other	10 (25%)
Medical (renal failure, COPD, sepsis, CVA, encephalitis, liver failure, hyponatremia, post-CPR)	22 (55%)
Surgical (trauma, upper GI bleeding, liver transplant, bowel perforation, necrotizing fasciitis)	18 (45%)

BMI, body mass index, WBC, white blood cells, COPD, chronic obstructive pulmonary disease, CVA, cerebrovascular accident, CPR, cardiopulmonary resuscitation, GI, gastrointestinal.

**Table 2:** Detailed telomer length change from first sampling to second sampling.

Patient Number	T/S Ratio of First Sampling	T/S Ratio of Second Sampling	Delta between First and Second Samplings
22	1	0.109	−0.891
24	1	1.855	0.855
25	1	0.305	−0.695
26	1	0.746	−0.254
27	1	0.505	−0.495
28	1	0.605	−0.395
29	1	1.485	0.485
30	1	1.237	0.237
32	1	0.704	-0.296
34	1	0.707	−0.293
35	1	1.618	0.618
37	1	0.785	−0.215
38	1	1.296	0.296
39	1	1.416	0.416
41	1	0.992	−0.008
45	1	0.626	−0.374
46	1	1.329	0.329
47	1	2.005	1.005
49	1	0.658	−0.342
50	1	1.6	0.6
51	1	0.902	−0.098
52	1	0.803	−0.197
53	1	0.937	−0.063
55	1	0.541	−0.459
56	1	0.608	−0.392
57	1	1.103	0.103
59	1	0.854	−0.146
60	1	0.82	−0.18
61	1	1.046	0.046
62	1	1.003	0.003
64	1	0.634	−0.366
65	1	0.867	−0.133
66	1	0.636	−0.364
68	1	0.622	−0.378
70	1	0.772	−0.228
71	1	0.594	−0.406
72	1	1.321	0.321
73	1	0.484	−0.516
74	1	1.5943	0.5943
75	1	0.824	−0.176

Delta, means the difference between two values.

**Table 3:** Patient characteristics and telomere length changes.

Patient Characteristic	Delta Telomere Length (>15% Baseline)			
	Shorter	Unchanged	Longer	*p*-Value
Number of patients	21	8	11	
Age (years ± SD)	45.38 *(±20.84)	49.50 (±15.63)	61.45 (±13.53)	0.0712
BMI (kg/m^2^ ± SD)	29.04 (±7.98)	28.35 (±8.12)	25.74 (±2.98)	0.4523
Male/Female	18/3	6/2	7/4	0.35813
APACHE-II score (±SD)	21 (±8)	24 (±5)	25 (±7)	0.2562
SOFA score	9 (±3)	10 (±3)	11 (±4)	0.3959
WBC count (K/mcL ± SD)	12.2 (±6.5)	11.6 (±5)	11.8 (±5.3)	0.9557
Platelet count (K/mcL ± SD)	203 (±94)	201 (±131)	156 (±76)	0.4128
CRP	16 (±13.3)	15.5 (±12.4)	11.6 (±13.1)	0.7066
Albumin	3 (±0.9)	2.8 (±0.9)	3 (±0.6)	0.8334
pH	7.3 (±0.11)	7.25 (±0.12)	7.31 (±0.11)	0.4361
Ventilated on admission	21/21	7/8	9/11	0.14956
Admission diagnosis				0.48791
trauma	9	2	3	
sepsis	6	1	1	
respiratory	3	1	1	
transplant	1	1	1	
other	2	3	5	
Medical/Surgical	10/11	5/3	7/4	0.61399

*, *p* value <0.05.

## Supplementary Materials

The following are available online at https://www.mdpi.com/2073-4425/10/10/761/s1, Table S1: Quality control with blood samples from healthy individuals.

## Abbreviations

**Abbreviation**	**Explanation**
ICU	intensive care unit
PCR	polymerase chain reaction
SIRS	systemic inflammatory response syndrome
DNA	deoxyribonucleic acid
CRP	c reactive protein
WBC	white blood cells
SOFA	sequential organ failure assessment
APACHE	Acute Physiology and Chronic Health Evaluation
RBC	red blood cells
USA	United States of America
SD	standard deviation
BMI	body mass index
COPD	chronic obstructive pulmonary disease
CVA	cerebrovascular accident
CPR	cardiopulmonary resuscitation
GI	gastrointestinal
PLT	platelets
FA	Fanconi anemia
UVA	ultraviolet A
ALT	alternative lengthening of telomeres
