# Supplementary material for: Telomere Length Changes during Critical Illness: A Prospective, Observational Study
Source: Genes (Basel). 2019 Sep 27;10(10):761. doi: 10.3390/genes10100761 (PMC6826589; doi:10.3390/genes10100761)
Supplement: Supplementary file 1 [file genes-10-00761-s001.docx]

Supplementary material
table-1 quality control with blood samples from healthy individuals

| **Δ between 1^st^ and 2^nd^ samplings** | **T/S ratio of 2^nd^ sampling** | **T/S ratio of 1^st^ sampling** | **Sample number** |
| --- | --- | --- | --- |
| 0.0004- | 1.2 | 1.2 | 1 |
| 0.0002 | 0.7 | 0.7 | 2 |
| 0.0001- | 0.6 | 0.6 | 3 |
| 0.0001- | 1 | 1 | 4 |
| 0.0004 | 1.1 | 1.1 | 5 |
| 0.0002 | 1.3 | 1.3 | 6 |
| 0.000 | 1.2 | 1.2 | 7 |
| 0.0002- | 1 | 1 | 8 |
| 0.0002 | 0.8 | 0.8 | 9 |
| 0.0001- | 0.9 | 0.9 | 10 |
